# Long-duration general anesthesia influences the intelligence of school age children

**DOI:** 10.1186/s12871-017-0462-8

**Published:** 2017-12-19

**Authors:** Qingqing Zhang, Yuanzhi Peng, Yingwei Wang

**Affiliations:** 1 Department of Anesthesiology and Critical Care Medicine, Xinhua Hospital Affiliated to Shanghai Jiaotong University, 1665 Kongjiang Road, Shanghai, China; 2 Department of Anesthesiology, Huashan Hospital Affiliated to Fudan University, 12 Wulumuqi Middle Road, Shanghai, 200040 China

**Keywords:** Children, Cognition, General anesthesia, Intelligence, Orthopedic surgery

## Abstract

**Background:**

General anesthesia has been linked to impaired brain development in immature animals and young children. In this study the influence of orthopedic surgery under general anesthesia on the intelligence of school age children has been evaluated.

**Methods:**

A total of 209 subjects aged 6–12 years were recruited and allocated into 4 groups according to the duration of general anesthesia, including a control group (*n* = 30), short (< 1 h, *n* = 49), moderate- (1–3 h, *n* = 51) and long-duration groups (> 3 h, *n* = 79), respectively. The intelligence quotient (IQ) of the subjects was measured by the Raven’s Standard Progressive Matrices (RSPM) before and after orthopedic surgery under general anesthesia of various durations (vide supra).

**Results:**

The IQ score decreased significantly in the long-duration group at 1 month post-operation compared with the pre-operation score (*P* < 0.001), and IQ did not recover completely at 3 months postoperatively (*P* < 0.05), but had recovered when measured at the 1-year follow-up. Moreover, this study showed that the development of children’s intelligence was affected by the exposure time to anesthetics at a younger age (OR = 5.26, 95% CI:2.70–8.41, *P* < 0.001), having a mother with a low education level (OR = 2.71, 95% CI:1.24–6.14, *P* = 0.014) and premature birth (OR = 2.76, 95% CI:1.34–5.46, *P* = 0.005).

**Conclusions:**

More than 3 h general anesthesia influenced the IQ of school age children for up to 3 months after orthopedic surgery. Beside extended exposure time to anesthetics additional factors for post-operative IQ reduction were younger children age, mothers with low educational levels and premature birth.

**Trial registration:**

Chinese Clinical Trial Registry with registration number ChiCTR-OOC-17013497 retrospectively registered on 11/23/2017.

## Background

General anesthesia induces a reversible unconscious state, allowing medical procedures to be performed without intolerable pain. However, previous studies in a various species of young animals have demonstrated that general anesthetics can cause neuronal cell death, impairment of learning and memory, alterations in dendritic morphology, and behavioral abnormalities [1–6]. It is generally considered that general anesthetics interfere with the pathways of neurotransmission that may be involved in the development of the central nervous system (CNS). Although the duration of exposure and doses administered in animals were not comparable to those administered to young children, the results of animal studies have raised serious concerns regarding the potential risk of anesthetics on pediatric neurological development.

Accumulating retrospective epidemiological studies have suggested a possible link between general anesthesia and cognitive function in children and it is generally believed that the effect of anesthetics on the developing brain is related to the extent of exposure and the age of children [7–10]. To minimize the differences between children with or without a medical history of general anesthesia, sibling studies were conducted to compare cognitive and academic impairments between pairs of twins. Although some studies found no differences between twins in which one had experienced general anesthesia and one had not [11], others showed that those who had undergone general anesthesia were more likely to have developmental or behavioral disorders [12]. A recent study has provided new evidence for a possible association between general anesthesia in young children with long-term diminution of language abilities and cognition, as well as regional volumetric alterations in brain structure [13]. Although it remains unresolved whether anesthesia itself is the cause, or that other unidentified factors associated with anesthesia are causally attributable to cognitive and behavioral problems in children, the potential side effects of general anesthesia for children is a major concern.

The majority of previous studies were retrospective and investigated children who had experienced anesthesia before the age of 3 or 4 years. Few studies have reported the effects of anesthesia in children who underwent surgery at school age. We assumed that the potential side effects of anesthetics on CNS development in young children might also be detectable at an older age. Therefore, the purpose of the present study was to assess the short- and long-term impact of general anesthesia after various durations of exposure on intellectual development and cognitive performance in school age children. The medical records and surgery procedures were carefully reviewed to avoid medical problems that were thought to be associated with vulnerabilities of child intelligence. Other potential risk factors were also analyzed to investigate the possible link of anesthesia exposure to the learning abilities of children who had experienced anesthesia and those who had not.

## Methods

### Participants

The current study was conducted in Shanghai Xinhua Hospital from 2009 to 2015. Based on preliminary tests, we estimated the sample size of our study to be at least 22 patients in each group (α = 0.05, β = 0.2, with a decreased intelligence score being the main variable). We enrolled 300 children aged 6–12 years who had scheduled orthopedic surgery under general anesthesia. Of these, 241 subjects were screened for pre-operative IQ measurement after excluding those who did not meet the inclusion criteria (*n* = 59) and those who refused to participate in the study (*n* = 7). Children were excluded from the study if they presented with physiological and/or psychological abnormalities during or after the operation (*n* = 32) or failed to complete 3 postoperative IQ tests as required (*n* = 32). Finally, we recruited 179 patients for analysis who were divided into the following 3 groups according to the duration of orthopedic surgery: short-duration (< 1 h, *n* = 49); moderate-duration (1–3 h, *n* = 51); and long-duration (≥ 3 h, *n* = 79). A total of 37 healthy volunteers with neither previous exposure to general anesthesia nor scheduled surgery were randomly enrolled from ordinary primary schools and those who completed 4 IQ measurement tests (*n* = 30) were used as the control group. The Ethical Committee of Shanghai Xinhua Hospital approved the study (No: XHEC-D-2009-018) and written informed consent was obtained from all participants’ guardians.

### Anesthesia and surgical procedures

Appropriate causes for surgery were selected for this study to minimize effects on post-operative intelligence as summarized in Table 1. The need for surgery was a single cause. Briefly, intravenous access was installed shortly before the induction of general anesthesia and endotracheal intubation. Vitals signs and the depth of anesthesia was monitored by electrocardiography (ECG) [14], pulse oxygen saturation (SPO_2_), end-tidal carbon dioxide tension (ETCO_2_), blood pressure, mean arterial pressure, and the bispectral index (BIS) during induction and maintenance of anesthesia. Analgesics were administered after surgery if required.

**Table 1:** Need for operation

Group	*n*	Need for Operation
Short-duration group	49	Supracondylar fracture of humerus, clavicle fracture, metatarsal and toe bone fractures
Moderate-duration group	51	Ulnar fracture, radius fracture, femoral fracture
Long-duration group	79	Compound fracture, dislocation of hip joint, chronic fracture healing deformity

The need for surgery was a single cause and the general anesthesia/surgical procedure was likely to have no or negligible effects on cognitive functions and the development of intelligence

### Intelligence assessment

All participants were tested using Raven Standard Progressive Matrices (RSPM) to measure intelligence and the level of mental development. The participant’s intelligence quotient (IQ) was obtained from the RSPM results for each appropriate age [15]. All children in the study had never previously had an RSPM assessment.

### Data collection

All participants were required to complete the RSPM tests independently in a quiet and comfortable place 1 day before, and 1 month, 3 months and 1 year after surgery. The calculated IQ scores were collected and computed by a qualified child psychologist and health professionals in the Mental Health of Children Department of Shanghai Xinhua Hospital.

Several potential confounding variables [10] which may interfere with interpreting an alteration of children cognition and behavior were recorded from the mother and father separately by the use of an additional questionnaire, including the child’s gestational age, age of exposure to general anesthesia, gender, mother’s education level, existing congenital disease and whether the child had experienced trauma within 1 year. The opinions of parents about any abnormalities of child intelligence and behavior before and after surgery were also recorded and evaluated.

### Statistical analysis

All data are shown as odds ratios (OR) and corresponding 95% confidence intervals (CIs). Statistical analyses were carried out using the Statistical Package for Social Science version 19.0 (SPSS Inc., Chicago, Illinois, USA) and two-tailed *P-*values <0.05 were considered to be statistically significant.

Differences of IQ scores at various time points within each group and among the 4 groups were analyzed by Friedman and Kruskal-Wallis tests, respectively. Multiple comparisons within each group and between the 4 groups were performed using a Dunn test. Multivariable logistic regression analysis was used to study the association between independent variables (confounding variables such as gender, gestational age, age of exposure to general anesthesia and a mother’s education level) and the dependent variable (IQ scores of children).

## Results

**Fig. 1:**
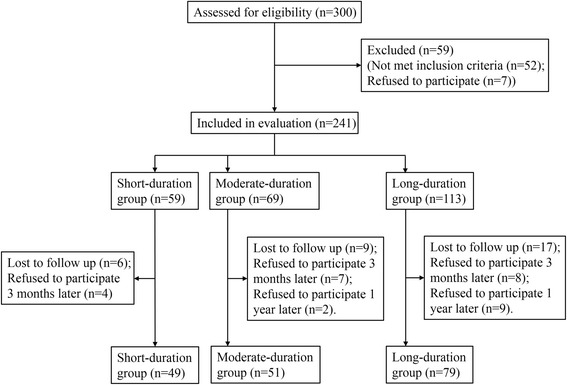
Flow diagram of patient enrollment, assignment, follow-up and analysis. A total of 179 subjects were recruited and allocated into various anesthesia exposure groups, namely short-duration (*n* = 49), moderate-duration (*n* = 51) and long-duration (*n* = 79 groups. In addition, 30 subjects served as the control group. Repeated measures analysis of variance (ANOVA) with mixed-effects modeling was used to analyze the differences, including those with incomplete follow-up data

Among 300 patients enrolled in the anesthesia exposure groups, a total of 64 children were excluded from the study due to physiological and/or psychological abnormalities during or after surgery (*n* = 32) or for failure to complete the required 4 RSPM tests (*n* = 6, 9 and 17 for the short-duration, moderate-duration and long-duration groups, respectively.). As shown in Fig. 1, a total of 179 pediatric patients (short duration group, *n* = 49), moderate duration group (*n* = 51) and long duration group (*n* = 79), and 30 healthy children (control group, *n* = 30, data not shown) completed the study.

**Fig. 2:**
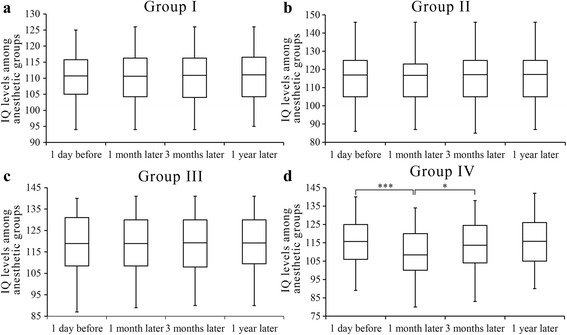
Comparison of the median intelligence levels after **b**) short (group II), **c**) intermediate (group III) or **d**) long term (group IV) anesthesia/surgery with **a**) group I as untreated controls at the indicated times before and after intervention. ***P < 0.001, *P < 0.05

Figure 2 shows all the medians and interquartile ranges of IQs calculated at pre- and post-operation times for each group. No significant difference was found between the pre-operative IQ in each anesthesia exposure group and the control group. In comparison with pre-operative IQ, there was no significant difference in the post-operative IQ obtained at 1 month, 3 months and 1 year in the control (group I), short (group II) and moderate (group III) duration groups.

However, the subjects in the long-duration group (group IV) had significantly compromised median intelligence levels at 1 month (*P* = 0.001), which did not recover completely until 3 months post-operation (*P* = 0.017). The incidence of intelligence reduction and behavioral abnormalities was increased at 1 and 3 months after surgical intervention compared with pre-operation levels (48.1% and 21.5%, respectively), while according to reviews and comments from parents only these children presented with decreased memorizing ability, logistical reasoning and judgment ability, as well as difficulty focusing on tasks or poor academic performance. No significant difference was found between pre-anesthesia and the 1 year post intervention IQs in all groups.

The cohort characteristics and potential confounders are listed in Table 2. We considered a decline of IQ > 5 points as being significant following guidance from a previous study [16]. No significant differences were found among the 4 groups at baseline.

**Table 2:** Characteristics and potential confounders of participants in each group

Characteristics	Entire cohort	Control group	Duration of general anesthesia		
			< 1 h	2–3 h	> 3 h
Number of subjects	209	30	49	51	79
Patient characteristics					
Boys, n (%)	107 (51.2)	15 (50.0)	26 (53.1)	26 (50.1)	40 (50.6)
Age, n (%)					
Younger (6-7 years old)	67 (32.1)	7 (23.3)	12 (24.5)	16 (31.4)	32 (40.5)
Middle (8–9 years old)	88 (42.1)	16 (53.3)	20 (40.8)	23 (45.1)	29 (36.7)
Older (10–12 years old)	54 (25.8)	7 (23.3)	17 (34.7)	12 (23.5)	18 (22.8)
Full-term pregnancy, n (%)	110 (52.6)	18 (60.0)	28 (57.1)	27 (52.9)	37 (46.8)
The education of mother					
Low (elementary school)	47 (22.5)	5 (16.7)	8 (16.3)	12 (23.5)	22 (27.8)
Middle (secondary school)	131 (62.7)	22 (73.3)	30 (61.2)	34 (66.7)	45 (57.0)
High (tertiary education)	31 (14.8)	3 (10.0)	11 (22.4)	5 (10.0)	12 (15.2)

Distribution of the participants’ characteristics and potential confounders are shown for the entire cohort and across the 4 groups of anesthetic exposure durations. Values represent *n* (%) for categorical variables

Table 3 summarizes the results of multivariable logistic regression analysis for potential confounding variables associated with alterations in a child’s IQ. Although no significant correlation was found between gender and the development of children’s intelligence, the risk was significantly increased for children who were young (OR = 5.26, 95% CI:2.70–8.41, *P* < 0.001), had a premature birth (OR = 2.76, 95% CI:1.34–5.46, *P* = 0.005) and had a mother with a low education level (OR = 2.71, 95% CI:1.24–6.14, *P* = 0.014).

**Table 3:** Results of logistic regression analysis of potentially related confounders to the dependent variable

Independent variable	Regression coefficient (β)	*P*-value	OR	95% CI for OR
Gender	−0.3136	0.433	0.731	[0.33, 1.60]
Age	−1.6901	< 0.001	5.26	[2.70, 8.41]
premature birth	−1.0174	0.005	2.76	[1.34, 5.46]
Mother’s education	−0.9962	0.014	2.71	[1.24, 6.14]

Note: CI, confidence interval

In comparison to children with poorly educated mothers, those with medium educated mothers appeared to be double, and compared with highly educated mothers four times less likely to have a compromised postoperative intelligence (percentage of postoperative intelligence impairment children from low (36.2%) vs medium (18.2%) vs highly (9.7%) educated mothers).

## Discussion

Multiple studies in young animals have demonstrated neurotoxicity and long-term learning impairment effects of general anesthetics that are commonly used in humans [1–6]. The possibility that anesthesia can affect intelligence and the learning ability of children has been a cause of concern over the past 10 years. Accordingly, the present study assessed the IQs of children aged 6–12 years before and after anesthesia/orthopedic surgery. Some important confounders that could affect the cognitive and behavioral development of children were also analyzed.

A number of studies have suggested a possible association between anesthesia and an increased risk of learning disabilities and behavioral problems in children who had experienced multiple or even a single episode of general anesthesia before the age of 3 years [7, 8, 10, 12]. A recent study suggested that children presented with slightly lower scores in listening comprehension and part of the IQ test, as well as slightly less gray matter in areas of their brain, after they had been exposed to general anesthesia before the age of 4 years [13]. In agreement with previous studies, our results demonstrated that the IQ of children aged 6–12 years significantly decreased at 1 and 3 months after long-duration exposure to general anesthesia, suggested similar potential neurological effects of anesthesia on children of school age, which is an important period of time in the overall neurodevelopment process. In addition, no significant differences in children’s intelligence and behavior were found after short- and moderate-duration exposure to anesthesia in our study, suggesting that the alteration of intelligence was associated with a single long exposure or a cumulative duration of anesthesia [7]. Moreover, it is generally believed that the growth spurt in cerebral development in humans commences at a gestational age of 6 weeks and lasts until three years after birth [8, 16]. The results of the recovery of intelligence at the 1 year follow-up of children exposed to long-duration anesthesia, and the increased risk for children who experienced anesthesia at a younger age (OR = 2.76, 95% CI 1.34–5.46, *P* = 0.005) suggests that older children are less susceptible to the neurological effects of general anesthetics compared with younger children.

In order to minimize confounding variables that may affect children’s neurodevelopment, multivariable logistic regression analysis was performed to identify potential risk factors associated with alteration of intelligence pre- and post-operation.

In addition to children’s exposure to anesthesia at a younger age, we found that the incidence of intelligence and behavior abnormalities were significantly increased in former preterm children who received long-duration anesthesia (Table 3). Previous studies have shown that premature infants were more susceptible to cognition and behavior deficits compared to term infants [17] and presented with more problems later at school age [18, 19]. Such handicaps can exist for circa 10 years [20] and a longer follow-up was recommended for preterm children [21]. According to our results, preterm children were more prone to post-operative temporary intelligence deficits after long-duration anaesthesia, which is underlined by previous findings that adverse sedation/anesthesia events are more common in former preterm children and might be the result of preexisting neurological conditions [22]. On the other hand, according to previous studies, preterm related developmental impairments can be avoided through early parental guidance and the educational level of mothers has been described as a factor for the school maturity of preterm children at the age of 8 [20, 21]. Different maternal attention to intellectual impairment by poorly educated mothers might also explain the finding of our study that their children were more likely to develop intellectual impairments after long-duration anesthesia (OR = 2.71, 95% CI 1.24–6.14, *P* = 0.014). However, further studies with a bigger cohort of patients will be required to elucidate further this important phenomenon.

Our study has several limitations: although the same anesthesia procedure was applied to every child to minimize potential influences of postoperative analgesics on cognitive abilities, we could not definitely distinguish the effects of anesthesia itself from other confounders associated with anesthesia. First, although we excluded surgeries (such as operations to treat obstructive sleep apnea syndrome [23] or nasosinusitis [24], urological and hepatorenal surgeries) which might have influenced postoperative intelligence, and pediatric patients with undiagnosed neurological complications observed at the 1 year follow-up after an orthopedic operation, it was difficult to conclude unequivocally it was the general anesthesia rather than the surgery per se that accounted for the change in intelligence [25], since patients might have experienced varying levels of stress due to surgical injury. Second, the lack of a standardized and valid methodology made it difficult to qualitatively and quantitatively evaluate childhood neurodevelopment in previous publications. Although RSMP is convenient, accurate and valid when used on repeated occasions [26], and was used to evaluate the development of children’s intelligence in our study, it was difficult to exclude completely false-negative measurements on learning effects. Finally, it should be kept in mind that it is still an open question whether exposure to general anesthesia is a potential or a causative agent leading to neurological impairment. Some sibling studies have suggested other factors such as genes or the home environment, other than anesthesia itself, might account for the differences in alteration of intelligent [11]. At present, only a few studies have investigated the effects of exposure to general anesthesia on children of school age. Therefore, further large-scale and pair-matched analysis will be needed to address the short- and long-term influences of exposure to general anesthesia on children of school age.

## Conclusion

More than 3 h general anesthesia during orthopedic surgery reduced the IQ for 3 months postoperatively. Additional factors for postoperative IQ reductions were youth, premature birth and a low educational level of the mother.

## Abbreviations

BIS: Bispectral index
CIs: Confidence intervals
CNS: Central nervous system
ECG: Electrocardiography
ETCO_2_: End-tidal carbon dioxide tension
IQ: Intelligence quotient
OR: Odds ratios
RSPM: Raven’s Standard Progressive Matrices
SPO_2_: Oxygen saturation
